# New molecular staging with G-factors (VEGF-C and Reg IV) by supplementing TNM classification in colorectal cancers

**DOI:** 10.3892/or.2013.2787

**Published:** 2013-10-03

**Authors:** TETSUJI SAWADA, MASAKAZU YASHIRO, KAZUHIRO SENTANI, NAOHIDE OUE, WATARU YASUI, KOHJI MIYAZAKI, KEITA KAI, HIDETO FUJITA, KEISHI NAKAMURA, KIYOSHI MAEDA, YOSHIHIRO KAKEJI, SHOJI NATSUGOE, KEN SHIRABE, SACHIYO NOMURA, YUTAKA SHIMADA, NAOHIRO TOMITA, KOSEI HIRAKAWA, YOSHIHIKO MAEHARA

**Affiliations:** 1 Department of Surgical Oncology, Osaka City University Graduate School of Medicine, Osaka, Japan; 2 Oncology Institute of Geriatrics and Medical Science, Osaka City University Graduate School of Medicine, Osaka, Japan; 3 Department of Molecular Pathology, Hiroshima University Institute of Biomedical and Health Sciences, Hiroshima, Japan; 4 Department of Surgery, Saga University Faculty of Medicine, Saga, Japan; 5 Department of Pathology and Microbiology, Saga University Faculty of Medicine, Saga, Japan; 6 Department of Gastroenterological Surgery, Division of Cancer Medicine, Graduate School of Medical Science, Kanazawa University, Kanazawa, Japan; 7 Division of Gastrointestinal Surgery, Department of Surgery, Graduate School of Medicine, Kobe University, Kobe, Japan; 8 Department of Surgical Oncology and Digestive Surgery, Kagoshima University School of Medicine, Kagoshima, Japan; 9 Department of Surgery and Science, Graduate School of Medical Sciences, Kyushu University, Fukuoka, Japan; 10 Department of Gastrointestinal Surgery, Graduate School of Medicine, University of Tokyo, Tokyo, Japan; 11 Department of Surgery and Science, Graduate School of Medicine and Pharmaceutical Sciences for Research, University of Toyama, Toyama, Japan; 12 Division of Lower GI, Department of Surgery, Hyogo College of Medicine, Hyogo, Japan

**Keywords:** colorectal cancer, G-factor, TNM classification, molecular stage, VEGF-C, Reg IV

## Abstract

Staging classification of colorectal cancers is performed by the UICC/TNM classification system, which is the global gold standard. However, we often experience in clinical practice that there are considerable differences in prognoses between patients who have the same classification particularly in stage II and III cancers. The aim of this study was to propose a new TNM-G classification to predict prognosis and recurrence by supplementing the conventional TNM classification. A total of 220 cases of colorectal cancer, including 77 at stage II and 143 at stage III, were registered from four independent facilities. Immunohistochemical staining for 7 molecules, such as p53, vascular endothelial growth factor (VEGF)-A, VEGF-C, regenerating islet-derived family, member 4 (Reg IV), olfactomedin 4, Claudin-18 and matrix metalloproteinase-7 (MMP-7), was performed to investigate the correlation between clinicopathological factors and expression of each molecule. Based on the results, no significant correlation was observed between the immunostaining expression of these 7 factors and recurrence in total colorectal cancer. Recurrence in stage II (77 cases) was significantly higher in cases positive for Reg IV expression (P=0.042). On analysis of overall survival (OS) and disease-free survival (DFS), VEGF-C and Reg IV expression had a correlation with poor prognosis, therefore, these factors were selected and applied to G-factor classifications so that cases negative for both could be classified as G0, cases positive for either of the factors could be classified as G1, and cases positive for both factors could be classified as G2. While no significant correlation was observed in the recurrence rates between G0 and G2, OS and DFS in stage II cases were significantly poorer for G2 cases in comparison with G0 or G1 cases. The survival curves of OS and DFS in stage II G2 were similar to that of stage III cases. According to these results, prognosis of VEGF-C/Reg IV both positive G2 cases in stage II colorectal cancer was found to be almost equal to the poor survival in stage III cases, and the advancement of one stage up migration based on G-factors may be supposed to be highly feasible for clinical application. In conclusion, the combination of VEGF-C and Reg IV may be a promising factor for clinical staging to supplement the classical TNM classification system, and it may suggest a good indication of adjuvant chemotherapy for G2 cases in stage II colorectal cancers.

## Introduction

The surgical treatment for colorectal cancer has been established, and the developed operating procedures in primary tumor resection with lymphadenectomy including laparoscopic technique have improved and prolonged the survival of colorectal cancer patients, particularly in stage II and III cases. To date, staging classification of colorectal cancers is performed by the UICC/TNM classification system (1) which is still used as the global gold standard for the decision in selecting treatment of cancers or predicting parameter for prognoses. However, we often experience in actual clinical practice that there are considerable differences in prognoses between patients who have the same classification particularly in stage III cancers, even though the advanced adjuvant chemotherapy after curative resection has been established. The adjuvant chemotherapy for stage II cases still remains controversial, because its benefit in survival has not been clearly defined. Therefore, the G-Project Committee was established by the Japan Society for Gastroenterological Carcinogenesis at the 2005 annual meeting with an aim of investigating whether a new TNM-G classification can be proposed to predict prognosis and recurrence by supplementing the conventional TNM classification. Gene expression (named as G-factors) which can transmit the molecular biological characteristics, would be included as prognostic factors and new classification of cancers in the TNM classification system. While evaluating the convenience of implementing the TNM-G classification system, it was determined that clinical application of RNA and DNA level analysis of the candidate factors would be challenging. Thus, analysis of protein expression levels by immunohistological staining of resected specimens was chosen for nominating the G-factors because of the relative ease of this method. Here, we conducted a multicenter collaborative study with cases extracted from several facilities.

## Materials and methods

### Patients

In total, 220 cases of colorectal cancer at stage II (n=77) and stage III (n=143) were registered from four institutions. Of 220 cases, 109 were confirmed postoperative recurrence or death within 5 years, and 111 cases were confirmed as 5–10 year recurrence-free survival. The pathological final stages were managed based on the UICC/TNM classification system (1). The four facilities, Department of Surgical Oncology, Osaka City University Graduate School of Medicine (Osaka, Japan); Department of Gastroenterological Surgery, Kanazawa University (Kanazawa, Japan); Department of Surgery and Science, Graduate School of Medicine, Kyushu University (Fukuoka, Japan); Department of Gastroenterological Surgery, Saga University, Faculty of Medicine (Saga, Japan), were selected to extract clinical cases, and resected specimens to examine evidence based on the correlation of staining outcomes and clinicopathologic factors. This study was conducted after obtaining approval from the society’s Ethics Committee at the annual meeting in 2007, and then requesting for approval from the ethics committee of each of the four facilities supplying resected specimens. Each facility provided samples according to an implementation planning report. The study protocol conformed to the ethical guidelines of the Declaration of Helsinki (1975). Upon gaining approval from the ethics committees, tissue samples were obtained from each specimen of the most recent cases from each facility, along with anonymous background data such as age, gender, occupation, operative procedure, degree of penetration into the wall (pT), lymph node metastasis (pN), final stage, ly and v factors, histological type, presence or absence of adjuvant therapy and regime, recurrence type, treatment after recurrence, postoperative disease-free survival (DFS), and postoperative overall survival (OS) period. The data and clinicopathological background factors were subsequently analyzed by the Department of Oncology at the Institute of Geriatrics and Medical Science, Graduate School of Medicine, Osaka City University.

### Selection for factor analysis and case extraction

As a preliminary step, a literature search of articles published between 1990 and 2005 was conducted in PubMed using the key word ‘colorectal cancer’ and ‘independent prognostic factors’. A total of 396 articles on colorectal cancer, were extracted and reviewed (Table I). The reports indicated 30 molecules as prognostic factors in colorectal cancer. These were classified into 9 groups based on molecular function: oncogenes, tumor suppressor genes, microsatellite instability, cell proliferation, growth factors/cytokines and their receptors, apoptosis signaling pathways, cell adhesion and invasion, angiogenesis, and others. Concerning the literature search, highly reported prognostic factors in colorectal cancers are: p53, 11 papers; microsatellite instability (MSI), 12; vascular endothelial growth factor (VEGF), 8; vascular density, 5; and CD44, 4. Based on these results, p53 and VEGF are the most common in colorectal cancers and supposed as candidate molecular factors namely ‘G factors’. Therefore, three factors, p53, VEGF-A and VEGF-C, were nominated as candidate factors (2–9) and evaluated in stage II and III cancers. In addition to the three factors, five molecules, regenerating islet-derived family, member 4 (Reg IV) (10,11), olfactomedin 4 (12), Claudin-18 (Invitrogen) (13) and matrix metalloproteinase-7 (MMP-7) (14), were added as candidate factors and evaluated.

### Methods of immunohistochemical staining and evaluation

In total, 110 cases of colorectal cancer, in which postoperative recurrence/death was confirmed within 5 years, and similarly, 110 cases, in which 5–10 year recurrence-free survival was confirmed, were used in a case-control study. A total of 220 patients who had undergone a R0 resection of the primary tumor and were confirmed histologically to have colorectal cancer, were enrolled in this study. Of the 220 cancers, 60 cases were from Osaka City University, 40 from Kanazawa University, 40 from Kyushu University, and 80 were from Saga University. The pathological final stages were managed based on Japanese Classification of Colorectal Carcinoma (7th edition) (15) which was revised based on the UICC/TNM Classification of malignant tumors (1). The above four institutions ultimately registered 220 cases of colorectal cancer (111 recurrence-free and 109 with recurrence), and these specimens were formalin-fixed and paraffin-embedded. Immunohistochemical staining was performed at the Department of Molecular Pathology at Hiroshima University (Hiroshima, Japan) using seven primary antibodies for p53 (DO-7; Dako), VEGF-A (Santa Cruz Biotechnologies, Inc.), and VEGF-C (American Research Products, Inc.), Reg IV; olfactomedin 4; Claudin-18 (Invitrogen); and MMP-7 (141-7B2; Daiichi Fine Chemicals, Inc.). Paraffin-embedded specimens were sectioned at 4 μm, hydrophilized, and microwaved for 30 min in pH 6.0 citric acid buffer or autoclaved in ethylenediaminetetraacetic acid buffer to activate the antigen. Intrinsic peroxidase was deactivated by incubation with 3% H_2_O_2_ for 10 min, and blocking was performed using sheep serum and reacting with each primary antibody for 1 h at room temperature. The samples were incubated in diaminobenzidine solution for 10 min, and counterstained with hematoxylin. The stained area was scored by the percentage of immunopositive cells as an index of the expression of each molecule. Cases that were not at all stained were scored as 0, cases with <10% of stained tumor cells were 1+, cases with 10–50% of stained tumor cells were 2+, and cases with >50% of stained tumor cells were 3+. Evaluation of immunostaining was conducted independently by two pathologists, and any discrepancies in assessment were discussed and reassessed by microscopy.

### Data analysis and testing for significant difference

The correlation between a clinicopathological factor and immunostaining result was analyzed by the Chi-square test or Fisher’s exact test. The survival duration was calculated using the Kaplan-Meier method and analyzed by the log-rank test to compare the cumulative survival durations in the patient groups. In all tests, a P-value of <0.05 was considered to represent statistical significance. SPSS statistical software (SPSS Japan Inc., Tokyo, Japan) was used for all analyses.

## Results

### Positive staining rate in colorectal cancer

**Figure 1:**
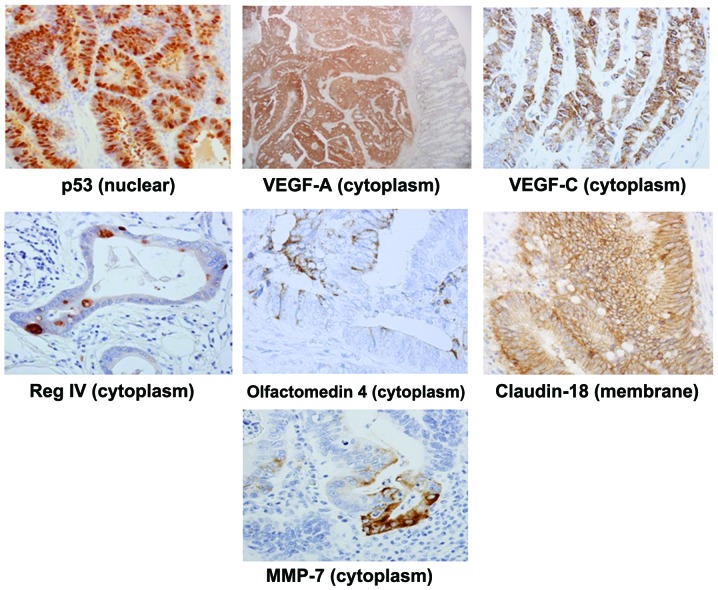
Immunohistochemical determination of p53, VEGF-A, VEGF-C, Reg IV, olfactomedin 4, Claudin-18 and MMP-7. p53 was found in the nuclei of cancer cells. Claudin-18 was observed at cell-cell boundaries of cancer cells. Other molecules were found in the cytoplasm of cancer cells.

Cases with >10% of stained tumor cells and scoring 2 or 3+ were assessed to be positive by two independent pathologists. Each representative positive expression in histological image for colorectal cancer is depicted in Fig. 1. Concerning to the positive staining rate of each factor, 51.8% of p53, 59.1% of VEGF-A, 60.0% of VEGF-C, 20.9% of Reg IV, 62.3% of olfactomedin 4, 8.2% of Claudin-18 and 45.5% of MMP-7 were positive in colorectal cancers (Table II).

### Correlation of postoperative recurrence and clinicopathological factors or the candidate molecular factors in colorectal cancer

Examination of the 220 colorectal cancer cases revealed a significant correlation between the postoperative recurrence and pT stage (P=0.008), pN stage (P=0.046), clinical stage (P=0.043), and ly factors (P=0.041), whereas no significant correlation was observed between the presence or absence of expression of the seven molecular factors and recurrence (Table II). Analysis of each stage revealed that the postoperative recurrence was significantly higher in Reg IV positive cases (P=0.042) at stage II in compared to negative cases, while no significant correlation was observed for any of the factors in stage III (Table III).

### Prognostic analysis of OS and DFS in expression of the candidate molecular factors of colorectal cancer

**Figure 2:**
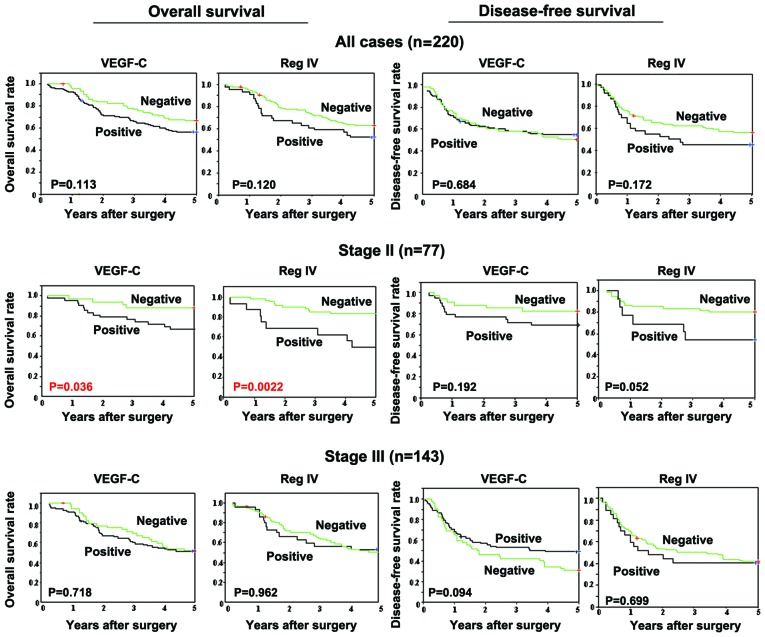
The overall (OS) and disease-free survival (DFS) curves of 220 colorectal cancer cases. Colorectal cancer with VEGF-C-positive expression showed a significantly worse OS time (P=0.036), and cases with Reg IV expression tend to have the worst OS time (P=0.0022).

In OS of stage II and III, colorectal cancer cases positive for VEGF-C and Reg IV tended to have poorer OS in comparison with the negative cases, although this was not significant. The prognosis of OS was significantly poorer (P=0.036) in stage II cases positive for VEGF-C expression in comparison with VEGF-C negative cases, moreover positive cases for Reg IV in stage II demonstrated significant poorer prognosis (P=0.0022) compared to negative cases. Reg IV positive cases at stage II and VEGF-C positive cases at stage III tended to have poorer DFS (P=0.052 and 0.094, respectively) (Fig. 2). In contrast, no significant difference was observed in OS between positive and negative cases for any of the 7 factors at stage III. Also, no significant difference of DFS was found between positive and negative groups in stage II cases.

### Feasibility of the candidate molecular factors

**Figure 3:**
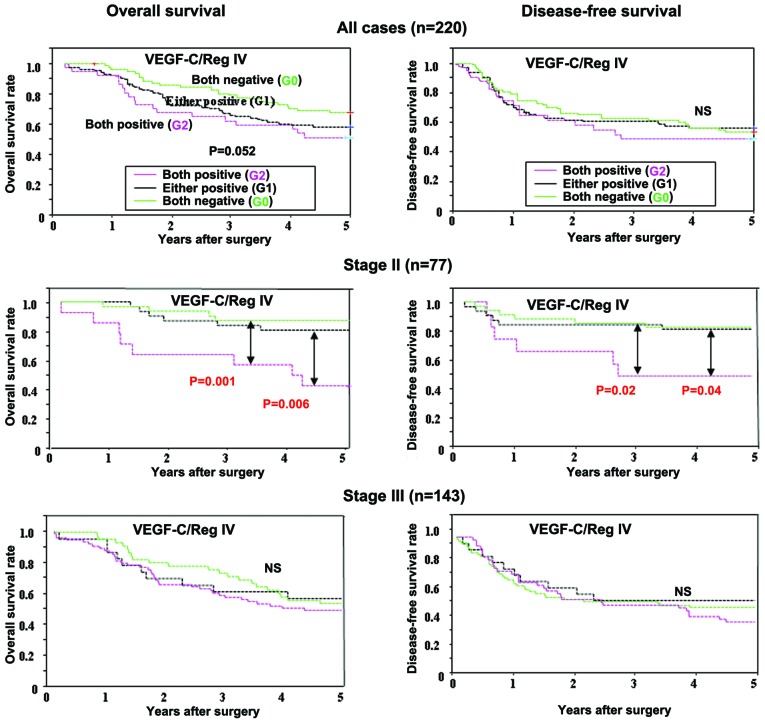
Survival of patients with colorectal cancer based on VEGF-C and Reg IV expression. In patients at stage II, the overall survival (OS) of the combination of VEGF-C and Reg IV positive group (G2) was significantly poorer than that of the combination of either positive (G1) and both negative group (G0) (P=0.006 and 0.001, respectively). The disease-free survival (DFS) of the combination of both VEGF-C and Reg IV positive group (G2) was significantly poorer than that of either positive (G1) and both negative group (G0) (P=0.02 and 0.04, respectively). In contrast, no significant difference of OS or DFS was observed among G0, G1, and G2 groups in all of cases or in stage III cases.

According to the above results, we selected VEGF-C and Reg IV as nominating factors in colorectal cancer. We then analyzed the relationship between the combination of VEGF-C and Reg IV expression and prognosis. Then, colorectal cancer patients were divided into three groups based on the VEGF-C and Reg IV expression; G0 group (both negative group, n=69), G1 group (either positive group, n=104), G2 group (both positive group, n=37). Fig. 3 shows the relationship between the combination of VEGF-C and Reg IV expression and prognosis. In stage II cases, OS of G2 cases were significantly poorer in comparison with that of G0 cases (P=0.001) and G1 cases (P=0.006), and DFS was also poorer than that of G0 cases (P=0.02) and G1 cases (P=0.04). In contrast, no significant difference of OS or DFS was observed among G0, G1 and G2 groups in all of cases or in stage III cases (Fig. 3). Table IV shows the relationship between the combination of VEGF-C and Reg IV expression and the postoperative recurrence. In all 220 colorectal cancer cases, the recurrence rate was slightly higher (59%) in G2 cases compared to 48% in G0 cases while no significant difference was observed (P=0.413). In stage II cases, the recurrence rate of G2 cases (64%) was high in comparison with that of G1 cases and G0 cases (32 and 37%, respectively), while the difference was not significant (P=0.117).

## Discussion

This study was a multicenter collaborative study with cases extracted from four universities. The four institutions ultimately registered 220 cases of colorectal cancer. The analysis of protein expression levels by immunohistological staining was selected for nominating the G-factors. The immunohistological staining was performed in each Department of Pathology by keeping patient infomation and clinicopathologic factors anonymous. The relationship between protein expression levels and clinicopathological background factors was independently analyzed at each facility. Therefore, the objectivity of these results can be considered to have high reliability and authenticity.

Precise clinical classification of prognosis might be useful to select a strategy for rigorous adjuvant chemotherapy and careful follow-up (16,17). The present study was conducted to establish a new classification system based on the biochemical characteristics of cancer, which would supplement the conventional TNM staging system. As shown in Fig. 2, VEGF-C and Reg IV expression was associated with a significantly poorer prognosis for OS of stage II colorectal cancer. However, these factors alone could not be found in the progressing stage to advance. Concerning these results, single use of G-factors by supplementing TNM staging may be difficult and limited. Because TNM staging is classified into T1-4, N0-3, and M0-1, consequently, in regard to the feasibility of TNM-G staging, combination of VEGF-C and Reg IV in 7 factors were selected and analyzed for usefulness. High frequent recurrences in stage II cases were observed in both positive cases (G2), but no relationship of recurrence was found among the 3 groups, G0-2. In contrast, a significant difference in OS was observed between G0/1 and G2 in stage II cases. Furthermore, the prognosis of OS and DFS of stage II G2 cases showed a survival curve apparently similar to that of stage III cases. This result indicated that prognosis of VEGF-C and Reg IV both positive G2 in stage II colorectal cancer was the same as that of stage III cases, and the advancement of one stage up based on G-factors may be highly feasible for clinical application. These findings suggested that TNM-G staging may have a possibility for use as a reasonable supplement to the TNM classification system.

Through the collaboration of many facilities and the Japan Society for Gastroenterological Carcinogenesis, the present study was schemed to investigate the feasibility of new molecular staging as a G-factor to further supplement the TNM classification system, which is the standard staging system used for colorectal cancers. With the advancement of molecular-targeting drugs, we investigated the possible application of G-factors, which are derived from molecular biological characteristics of cancer, in staging along with clinicopathological factors. However, in accordance with previous reports from single institutions, no correlation between recurrence/prognosis and up-staging migration was found. Thus, the proposal of an individual single G-factor was supposed to be challenging. However, when colorectal cancer was limited to stage II, the present results indicated that G2 cases both positive for VEGF-C/Reg IV were likely to advance up to stage III, suggesting that G-factors can be used to supplement initial staging by TNM classification. Application and effect of adjuvant chemotherapy for stage II colorectal cancers still remains controversial. The present results may suggest a good indication of adjuvant chemotherapy for G2 cases in stage II. In future studies, the highly relevant factors may be identified by the involvement of the degree of molecular biological malignancy to establish TNM-G staging, and application of these factors by supplementing TNM classification may contribute to more accurate prediction of prognosis.

In conclusion, the members of the Japan Society for Gastroenterological Carcinogenesis, investigated the feasibility of a new molecular factor(s) to further supplement the TNM classification system, and found that the combination of VEGF-C and Reg IV might be a promising factor for clinical staging to supplement the classical TNM classification system, and it may suggest a good indication of adjuvant chemotherapy for G2 cases in stage II colorectal cancers.

## Figures and Tables

**Table I:** Prognostic factors in colorectal cancer.

Category	Molecules	No. of papers by multivariate (M) or univariate (U) analysis
Oncogene	*k-ras*	
	*c-erbB-2*	
Tumor suppressor gene	*P53*	M, 6^a^; U, 5^b^
	*DCC*	
	*SPN*	
MSI (MMR gene)	MSI/BAT	M, 4^a^; U, 8^b^
Cell proliferation	Polyamine	
Growth factor/cytokine and those receptor	VEGF	M, 2^a^; U, 6^b^
	IL6	
	IGF	
	cMet	
	EGFR	
Apoptosis signal pathway	TRAIL	
Cell invasion and adhesion	MRP-1	
	uPA	
	Matrilysin	
	S100A4	
	Angiomodulin	
	CD44	M, 1^a^; U, 3^b^
	Laminin	
	β6-integrin	
	α3-integrin	
Angiogenesis	CD105	
Others	Vascular density	M, 3^a^; U, 2^b^
	Galectin	
	CD95	
	Telomerase	

a No. of papers by multivariate analysis;

b no. of papers by univariate analysis. The prognostic factors reported in the 396 published articles between 1990–2005.

**Table II:** Correlation between postoperative recurrence and clinicopathological features in 220 patients with colorectal cancer.

	Recurrence		
		
Clinicopathological factors	Negative n=111 (50%)	Positive n=109 (50%)	P-value
Gender			
Male	59 (47)	66 (53)	0.268
Female	52 (55)	43 (45)	
Location			
Right	31 (54)	26 (46)	0.465
Left	79 (49)	83 (51)	
pT			
1	2 (100)	0 (0)	0.008
2	5 (50)	5 (50)	
3	96 (53)	84 (47)	
4	8 (29)	20 (71)	
pN			
Negative	48 (59)	33 (41)	0.046
Positive	63 (45)	76 (55)	
Stage			
II	46 (60)	31 (40)	0.043
III	65 (46)	78 (54)	
Histologic type			
Diffuse	11 (38)	18 (62)	0.140
Intestinal	100 (53)	90 (47)	
Lymphatic invasion			
Negative	53 (60)	35 (40)	0.041
Positive	58 (44)	73 (56)	
Venous invasion			
Negative	74 (52)	68 (48)	0.509
Positive	37 (48)	40 (52)	
p53			
Negative	51 (48)	55 (52)	0.503
Positive	60 (53)	54 (47)	
VEGF-A			
Negative	46 (51)	44 (49)	0.871
Positive	65 (50)	65 (50)	
VEGF-C			
Negative	46 (52)	42 (48)	0.660
Positive	65 (49)	67 (51)	
Reg IV			
Negative	91 (52)	83 (48)	0.287
Positive	20 (44)	26 (56)	
Olfactomedin 4			
Negative	43 (52)	40 (48)	0.755
Positive	68 (50)	69 (50)	
Claudin18			
Negative	104 (52)	98 (48)	0.306
Positive	7 (39)	11 (61)	
MMP-7			
Negative	58 (48)	62 (52)	0.491
Positive	53 (53)	47 (47)	

**Table III:** Correlation between postoperative recurrence and candidate G-factors in 220 patients with colorectal carcinoma at stage II and III.

	Stage II (n=77) Recurrence			Stage III (n=143) Recurrence		
		
Clinicopathological factors	Negative n=46 (60%)	Positive n=31 (40%)	P-value	Negative n=65 (45%)	Positive n=78 (55%)	P-value
p53						
Negative	17 (53)	15 (47)	0.318	34 (46)	40 (54)	0.903
Positive	29 (64)	16 (36)		31 (45)	38 (55)	
VEGF-A						
Negative	18 (60)	12 (40)	0.970	28 (47)	32 (53)	0.805
Positive	28 (60)	19 (40)		37 (45)	46 (55)	
VEGF-C						
Negative	21 (62)	13 (38)	0.747	25 (46)	29 (54)	0.875
Positive	25 (58)	18 (42)		40 (45)	49 (55)	
Reg IV						
Negative	40 (66)	21 (34)	0.042	51 (45)	62 (55)	0.881
Positive	6 (38)	10 (62)		14 (47)	16 (53)	
Olfactomedin 4						
Negative	19 (68)	9 (32)	0.272	24 (44)	31 (56)	0.730
Positive	27 (55)	22 (45)		41 (47)	47 (53)	
Claudin-18						
Negative	43 (61)	28 (39)	0.612	61 (47)	70 (53)	0.378
Positive	3 (50)	3 (50)		4 (33)	8 (67)	
MMP-7						
Negative	23 (55)	19 (45)	0.329	35 (45)	43 (55)	0.878
Positive	23 (66)	12 (34)		30 (46)	35 (54)	

**Table IV:** Relationship between reccurrence and two molecular factors.

	Recurrence, n (%)		
		
VEGF-C and Reg IV	Negative	Positive	P-value
Total (n=220)	111 (51)	109 (49)	
Both negative G0 (n=79)	41 (52)	38 (48)	
Either positive G1 (n=104)	55 (53)	49 (47)	
Both positive G2 (n=37)	15 (41)	22 (59)	0.413
Stage II (n=77)	46 (60)	31 (40)	
Both negative G0 (n=32)	20 (63)	12 (37)	
Either positive G1 (n=31)	21 (68)	10 (32)	
Both positive G2 (n=14)	5 (36)	9 (64)	0.117
Stage III (n=143)	65 (45)	78 (55)	
Both negative G0 (n=47)	21 (45)	26 (55)	
Either positive G1 (n=73)	34 (47)	39 (53)	
Both positive G2 (n=23)	10 (43)	13 (57)	0.959
